# Cellular Mechanisms of Action of Drug Abuse on Olfactory Neurons

**DOI:** 10.3390/ijerph13010005

**Published:** 2015-12-22

**Authors:** Thomas Heinbockel, Ze-Jun Wang

**Affiliations:** Department of Anatomy, College of Medicine, Howard University, Washington, DC 20059, USA; zejunwang@hotmail.com

**Keywords:** brain, cannabinoid, central nervous system, drug abuse, drug addiction, electrophysiology, health disparity, marijuana, neuroscience, olfaction

## Abstract

Cannabinoids (Δ9-tetrahydrocannabinol) are the active ingredient of marijuana (cannabis) which is the most commonly abused illicit drug in the USA. In addition to being known and used as recreational drugs, cannabinoids are produced endogenously by neurons in the brain (endocannabinoids) and serve as important signaling molecules in the nervous system and the rest of the body. Cannabinoids have been implicated in bodily processes both in health and disease. Recent pharmacological and physiological experiments have described novel aspects of classic brain signaling mechanisms or revealed unknown mechanisms of cellular communication involving the endocannabinoid system. While several forms of signaling have been described for endocannabinoids, the most distinguishing feature of endocannabinoids is their ability to act as retrograde messengers in neural circuits. Neurons in the main olfactory bulb express high levels of cannabinoid receptors. Here, we describe the cellular mechanisms and function of this novel brain signaling system in regulating neural activity at synapses in olfactory circuits. Results from basic research have the potential to provide the groundwork for translating the neurobiology of drug abuse to the realm of the pharmacotherapeutic treatment of addiction, specifically marijuana substance use disorder.

## 1. Introduction

Drug addiction is a brain disease that afflicts millions of individuals in the USA, particularly individuals in minority populations ([1]; CDC OMHD website). Drug addiction costs society enormously in terms of medical and social expense. The amount of suffering among those with the disease and their loved ones is tremendous. Drugs tamper with brain circuits either through imitating the brain’s own chemical messengers or by over stimulating reward circuits in the brain such that an addicted person has a strong urge to use a drug and cannot stop, even if they want to [1]. One aim of drug addiction research is to understand the neurobiological mechanisms of this disease. Results from basic research are designed to help in the development of treatment strategies to prevent drug abuse, e.g., marijuana substance use disorder, and to eliminate health disparities. Substance use disorder indicates that a person needs a drug to function normally, and prevention of drug use leads to withdrawal symptoms. This paper focuses on the cellular actions of cannabinoids (CBs) on nerve cells to determine the fundamental biological mechanisms involved in a disease condition that disproportionately affects racial/ethnic minority populations and health disparity populations. According to the Center for Disease Control and Prevention, Office of Minority Health and Health Disparities (CDC OMHD), race and ethnicity correlate with persistent, and often increasing, health disparities among U.S. populations (website: http://www.cdc.gov/omhd; Center for Disease Control and Prevention, Office of Minority Health and Health Disparities). In the foreseeable future, racial and ethnic minority groups will constitute an increasingly larger proportion of the U.S. population. Therefore, the health of America will be significantly impacted by our efforts and success in improving the health of these groups. While great strides have been made to improve the overall health of the nation, Americans who are members of racial and ethnic minority groups, including blacks or African Americans, American Indians, and Alaska Natives, Asian Americans, Hispanics or Latinos, and Other Pacific Islanders, are more likely than whites to have poor health and to die prematurely (CDC OMHD). Health disparities are thought to reflect complex interactions among genetic variations, environmental factors, and specific health behaviors.

## 2. The Endocannabinoid System

The endogenous cannabinoid system (endocannabinoid system, endoCB system) was first discovered because it can be activated by a plant-derived compound—In the case of the endoCBs this is Δ9-tetrahydrocannabinol, THC, the bioactive ingredient of the drugs marijuana and hashish [2]. The resemblance between marijuana and endoCBs allows marijuana, *i.e.*, THC, to activate CB receptors. It is important to point out that endoCBs rather than marijuana evolved together with CB receptors to serve as a brain communication system. THC happens to bind to the same receptors, CB receptors, as brain-produced endoCBs. The endoCB system (CB receptors, and their ligands, CBs) has important intrinsic roles as a neuromodulator during normal brain function. CBs are produced endogenously by neurons in the brain (endoCBs) and serve as important signaling molecules in the nervous system and the rest of the body [3,4,5,6,7,8,9,10]. CBs are important in many bodily processes both in health and disease [11,12,13,14], in vertebrates and invertebrates [15]. Recent pharmacological and physiological experiments have described novel aspects of classic brain signaling mechanisms or revealed unknown mechanisms of cellular communication involving the endoCB system [9,16,17]. While several forms of signaling have been described for endoCBs [17], the most distinguishing feature of endoCBs is their ability to act as retrograde messengers in neural circuits. A recent example from the olfactory system illustrates this signaling cascade [18] and is described in this review.

Chemically, endoCBs are small lipids that regulate various aspects of brain function such as learning and memory, synaptic transmission and plasticity as well as growth and development [4]. Two endoCBs, N-arachidonoylethanol-amide (anandamide, AEA) and 2-arachidonoylglycerol (2-AG) are the principal natural agonists/ligands of the most widely expressed CB receptor in the brain, cannabinoid receptor 1, CB1R [19]. These two endoCBs, anandamide and 2-AG, are produced in the brain, bind to CB1R and have the same functional activity as marijuana [2]. The similarity between THC and endoCBs allows THC to activate the brain CB signaling system which originally evolved with endogenously produced CBs binding and activating CB1R. Other minor lipid metabolites different from, but chemically similar to, anandamide and and 2-AG have been suggested to act as endoCBs [20].

In addition to the fatty-acid derived endogenous ligands, the endoCBs, the endoCB system comprises G-protein coupled CB receptors, as well as the associated biochemical machinery with endoCB precursors, synthetic and degradative enzymes for these lipid neurotransmitters, and transporters [3,5,7,8]. Two different CB receptors exist, CB1 and CB2 receptors (CB1R, CB2R), with 44% amino acid sequence homology [21,22]. In the brain, CB1R is the most abundant G-protein coupled receptor [23]. CB2R is primarily expressed in immune cells and peripheral tissues [22] even though some level of CB2R expression has been detected in the brainstem, cortex, and cerebellar neurons and microglia [24,25]. CB1R is found in all normal brains [21,23,26] and has many essential brain functions when activated by their natural ligands. EndoCBs are synthesized from membrane lipids and act as the endogenous ligands for G_i/o_-protein-coupled type 1 CB receptors (CB1Rs) [27]. They can diffuse through membranes and are able to activate CB receptors in the same manner as exogenous CBs, such as THC [28]. EndoCBs are produced and released mainly “on demand” [5]. After release and binding to CB1R, they are rapidly cleared from the extracellular space by a process of cellular uptake followed by metabolism [29].

## 3. Retrograde Signaling with Endocannabinoids

**Figure 1:**
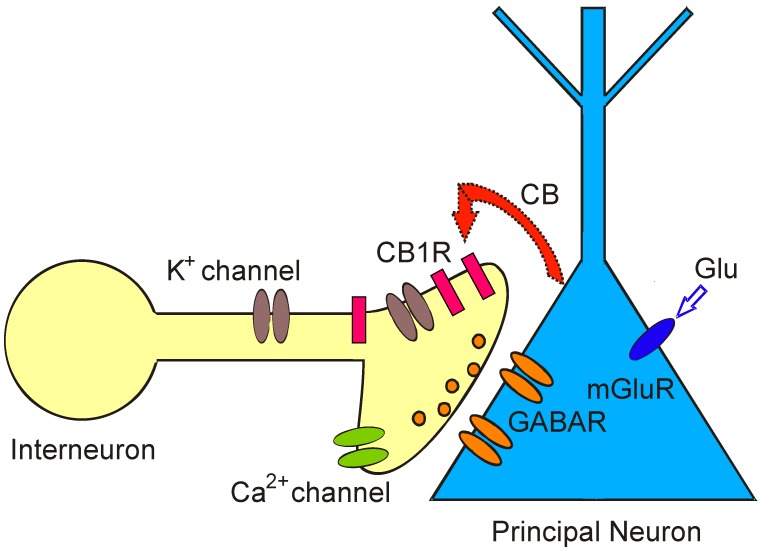
Depolarization-induced Suppression of Inhibition (DSI) is a model for retrograde signaling in the brain and allows assaying real time release of endoCBs from principal neurons as a brief cessation of GABA ouput. Activation of metabotropic glutamate receptors (mGluRs) by glutamate (Glu) on principal neurons or depolarization of postsynaptic principal cells evokes synthesis and release of cannabinoids (CB). Cannabinoids bind to presynaptic cannabinoid receptors (CB1R) on GABAergic interneurons and transiently reduce GABA release from synaptic terminals. As a consequence, GABA_A_ receptor-mediated synaptic currents and GABAergic inhibition are temporarily suppressed in postsynaptic principal neurons.

Some fifteen years ago, endoCBs were found to be unconventional neurotransmitters. In contrast to conventional neurotransmitters that are synaptically released from presynaptic neurons and bind to receptors on postsynaptic neurons, endoCBs are lipids and can act as retrograde signaling molecules that are released non-synaptically anywhere from activated neurons. Their retrograde signaling mode has been described in the hippocampus [3,19,30,31,32,33,34,35], cerebellum [36,37,38], neocortex [39,40], amygdala [41,42], and olfactory bulb [18]. EndoCBs are not stored intracellularly but are rapidly synthesized from components of the cell membrane and released from neurons when intracellular calcium levels rise or in response to activation of certain G-protein-coupled receptors. A brief rise in intracellular calcium concentration inside a pyramidal cell of the hippocampus results in a decline of incoming GABAergic inhibitory signals from presynaptic neurons. After release, endoCBs act as CB1Rs on nearby presynaptic terminals to reduce neurotransmitter release (GABA). The observation of this physiological response leads to the description of a type of short-term synaptic plasticity, originally observed in the cerebellum and hippocampus and mediated by endoCBs, namely DSI (Depolarization-induced Suppression of Inhibition) (Figure 1). In DSI, endoCBs are released from depolarized principal neurons and travel to presynaptic inhibitory interneurons to transiently reduce presynaptic firing and neurotransmitter (GABA) release [2,3]. Since endoCBs are fat-soluble molecules, they do not diffuse over great distances in the watery extracellular environment of the brain. Instead, DSI acts as a short-lived local effect that enables individual neurons to disconnect briefly from their neighbors and encode information [3]. During DSI, neurons control their own synaptic excitability in an activity-dependent manner and are able to alter the strength of synapses made onto them through. DSI is functionally relevant in information processing by neuronal networks [7]. In the cerebellum, a retrograde signaling process similar to DSI reduces synaptic excitation by suppressing presynaptic glutamate release and is called “DSE” [43].

## 4. Organization of the Main Olfactory Bulb

**Figure 2:**
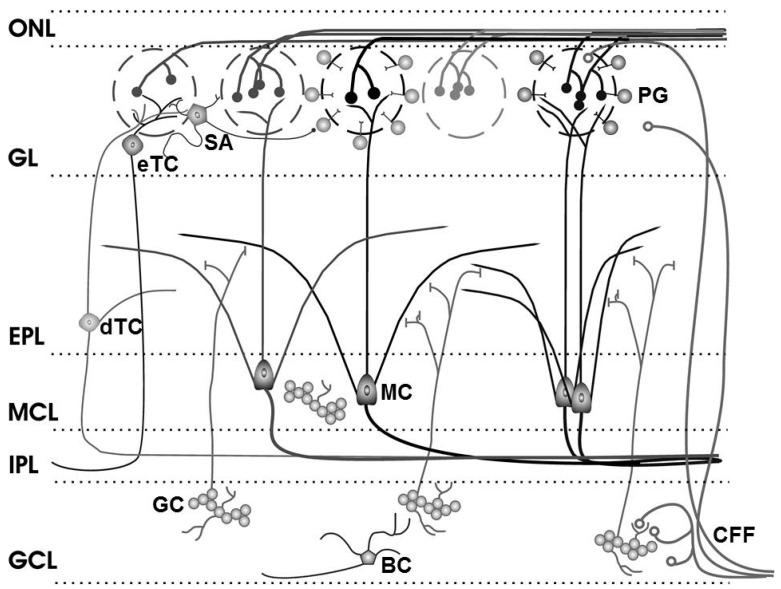
Olfactory bulb circuitry. Olfactory receptor neuron axons enter the main olfactory bulb through the olfactory nerve laver (ONL), to synapse with periglomerular cells (PG), mitral cells (MC) and tufted cells (of which external, eTC, and deep, dTC, tufted cells are shown) within the glomerular layer (GL). Short Axon (SA) cell axons receive synaptic input from eTCs and form extensive interconnections between glomeruli, while mitral cell apical dendrites convey sensory information to deeper layers of the bulb. In the external plexiform layer (EPL), mitral and (deep) tufted cells extend lateral dendrites which release glutamate onto the dendrites of granule cells (GC). Mitral cell bodies are located in the mitral cell layer (MCL), which is also densely packed with granule cells. Mitral and tufted cell axons project through the internal plexiform layer (IPL) to olfactory cortex (their axon collaterals branching into the GCL are not shown). The granule cell layer (GCL) contains the major population of inhibitory granule cells. Blane’s cells (BC) within the GCL make inhibitory contact with granule cells. Centrifugal fibers (CFF) shown projecting to the GL and GCL include glutamate-releasing axons of olfactory cortex pyramidal cells receiving mitral cell output. Modified from [60]; original drawing by Cristina Shirley.

Despite an increasing realization of the relevance of the endoCB system for numerous brain structures and human behavior, the role of this signaling system for odor processing largely awaits investigation [18,44]. The main olfactory bulb is the first relay station in the CNS for processing of sensory information that comes from olfactory receptor cells in the nasal epithelium. Synaptic processing in the main olfactory bulb is dominated by modulatory input. The relay from the nose to principal neurons in the main olfactory bulb, mitral, and tufted cells, and from mitral/tufted cells to higher order olfactory centers is strongly regulated by local intrabulbar circuitry, as well as centrifugal inputs to the main olfactory bulb from other brain areas (Figure 2). The cell bodies of different types of tufted cells are found in specific layers of the main olfactory bulb (glomerular layer: External tufted cells; external plexiform layer: Deep tufted cells). Mitral cells have their cell body in the mitral cell layer with an apical dendrite reaching into the glomerular layer and several lateral dendrites in the external plexiform layer. Both mitral and tufted cells integrate sensory and synaptic information that comes from either the olfactory epithelium in the nose or from the intrabulbar circuitry, *i.e.*, neurons within the main olfactory bulb. The intrabulbar circuitry includes GABAergic interneurons, such as periglomerular cells and granule cells [45]. These neurons have their cell bodies in the glomerular or granule cell layer, respectively. CB receptors are expressed at high levels in the main olfactory bulb, specifically in the input region, the glomerular layer [26,46,47,48]. Neurons in the glomerular layer are immunoreactive for enzymes that synthesize endoCBs [49,50,51]. Therefore, a pressing issue in the organization and operation of the olfactory system is the functional significance of modulatory input provided by the endoCB system.

The glomerular layer (see Figure 2: GL) houses the cell bodies of three neuronal subpopulations: periglomerular (PG), external tufted (eTC), and short-axon (SA) cells. The GABAergicperiglomerular cells are neurochemically and functionally heterogeneous [52,53,54]. The cell bodies of periglomerular cells are located at the periphery of the olfactory glomeruli in the glomerular layer, *i.e.*, the input layer of the main olfactory bulb. Short-axon cells express both GABA and dopamine, and external tufted cells are glutamatergic [52,55]. Input from the olfactory nerve targets periglomerular cells which also receive dendrodendriticglutamatergic input from external tufted or mitral cells, e.g., as spontaneous bursts of excitatory postsynaptic currents (EPSCs) [53,55,56]. Periglomerular cells mediate presynaptic inhibition of olfactory receptor neurons through GABAergic transmission [57,58]. External tufted cells are targeted by periglomerular cells that evoke spontaneous bursts of inhibitory postsynaptic currents (sIPSCs) at inhibitory GABAergic synapses with external tufted cells but they also receive spontaneous glutamatergic EPSCs [56,59].

## 5. Endocannabinoids in the Olfactory System

Recent work using patch-clamp electrophysiology in brain slices has established that the endoCB system plays a functional role in regulating neuronal activity and signaling in olfactory bulb glomeruli [18]. Specifically, CB receptors directly regulate membrane properties of periglomerular cells as shown by the effects of CB1R antagonist AM251 and agonist WIN (WIN55,212-2 mesylate) in the presence of ionotropic glutamate (NMDA and AMPA receptors) and GABA_A_ receptor blockers (synaptic blockers: CNQX to block AMPA receptors, APV to block NMDA receptors, gabazine to block GABA_A_ receptors). The actions of CBs on periglomerular cells are mediated through CB1R expressed by periglomerularcells. AM251 directly activates periglomerular cells and enhances their GABA release. Since periglomerular cells are synaptically connected to external tufted cells, any CB1R-mediated regulation of activity of periglomerular cells can affect GABA release and synaptic transmission to external tufted cells.

External tufted cells themselves express CB1R which may participate in modulating their activity. Neither AM251 nor WIN influences firing frequency or membrane potential in external tufted cells [18]. However, CB drugs can have a modest effect on external tufted cells when synaptic blockers prevent communication to other cells. In this condition, AM251 slightly increases the firing rate of external tufted cells without membrane depolarization and WIN slightly decreases their firing without a clear change in membrane potential. These effects indicate that during pharmacological isolation of external tufted cells CB1R mediates a direct effect on external tufted cells. The modest direct excitatory effect of a CB1R antagonist on external tufted cells is opposed by a much stronger effect, namely, increased GABAergic synaptic input from periglomerular cells onto external tufted cells. The enhanced GABA release from periglomerular cells triggered by a CB1R antagonist overshadows the CB1R antagonist-evoked direct excitation of external tufted cells.

**Figure 3:**
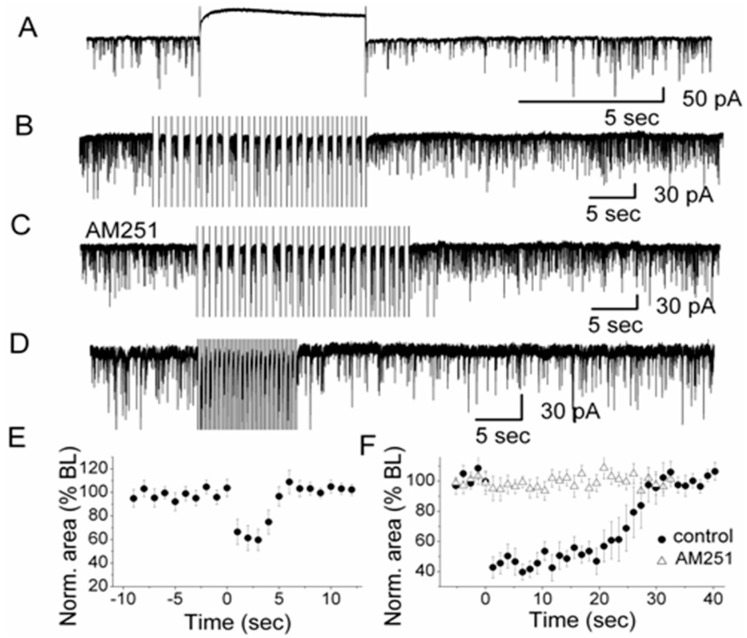
Depolarization-induced Suppression of Inhibition (DSI) in olfactory glomeruli. (**A**) A depolarizing voltage step (indicated by the 5-s silent trace in the recording) evoked DSI in a representative external tufted cell after the end of the voltage step. High Cl^−^-based pipette solution was used for recording sIPSCs. Depolarization was achieved by stepping from −60 mV holding potential to 0 mV for 5 s; (**B)** In the presence of CNQX and APV to block ionotropic glutamate receptors (NMDA and AMPA receptors), a train of 20 voltage steps to 0 mV (0.75 Hz; step duration: 667 ms) transiently reduced sIPSCs in an external tufted cell. Holding potential was −60 mV; (**C**) In the presence of CB1R antagonist AM251, no sIPSC suppression was observed, *i.e.*, DSI was mediated by CB1R; (**D**) A train of 20 voltage steps to −30 mV (2 Hz; step duration: 250 ms) which mimicked natural rhythmic bursting of external tufted cells, transiently reduced sIPSCs in an external tufted cell (in CNQX and APV); (**E**) Normalized sIPSCs area illustrating the magnitude and time course of DSI elicited by a 5-s depolarizing pulse (*n* = 7). The averaged values between 0–5 s after the end of the voltage step were significantly different from the baseline (ANOVA and Bonferroni post-hoc analysis, *p*< 0.05); (**F**) Normalized sIPSC area illustrating the magnitude and time course of DSI elicited by a train of depolarizations to 0 mV (*n* =12) in control and in the presence of AM251 (*n* = 10). In control conditions, the averaged values between zero to 25 s after the end of the train of voltage steps were significantly different from the baseline (ANOVA and Bonferroni post-hoc analysis, *p*< 0.05). From [18] with permission of the Society for Neuroscience.

Given the effects of CB1R on periglomerular and external tufted cells, the question arises if DSI is present in the glomerular layer of the olfactory bulb. Indeed, in external tufted cells, DSI can be induced with a 5-s depolarizing voltage step from a holding potential of −60 mV to 0 mV (Figure 3A). During DSI, sIPSCs are decreased in amplitude and frequency in these cells. As shown in Figure 3A, a single 5-s depolarizing voltage step which is visible in the trace as the period with no activity between the onset and end artifact, can reduce sIPSCs after the voltage step for several seconds. External tufted cells are characterized by a distinct intrinsic bursting pattern of action potentials [56]. This bursting pattern can be mimicked experimentally by applying a train of depolarizing steps to an external tufted cell. The experiment can also reveal a potential functional role of DSI in glomeruli. A train of depolarizing steps transiently suppresses sIPSC area to 60% of control (20 steps, 0.75 Hz) (Figure 3B). In order to test if DSI relies on activation of CB1R, the CB1R antagonist AM251 is present before and during the voltage steps (Figure 3C). DSI is indeed mediated by CB1R as shown by the fact that DSI can be completely eliminated in the presence of AM251 (Figure 3C). External tufted cells have an intrinsic bursting frequency ranging from 0.5 to 6.5 Hz with a mean frequency of 2.7 bursts/s [56]. A series of depolarizing voltage pulses at 2 Hz (20 steps, pulse duration: 250 ms) which mimics the intrinsic bursting, evokes DSI as a reduction of sIPSCs in external tufted cells (Figure 3D). The 5-s depolarizing voltage step can suppress the sIPSC area by ~40% of control followed by gradually recovery (Figure 3E). Single depolarizing voltage steps as well as a train of voltage steps (Figure 3F) evoke suppression of inhibition (DSI) in external tufted cells suggesting that spontaneous rhythmic bursting of these cells triggers the release of endoCBs. The releasedendoCBs function as retrograde messengers to reduce GABA release from periglomerular cells. This, in turn, regulates the activity of synaptic targets of periglomerular cells such as external tufted cells.

DSI occurs in external tufted cells. A train of depolarizing voltage steps (>3 steps) generates particularly prominent DSI in external tufted cells and strengthens the inhibition of sIPSCs. The naturally occurring rhythmic burst firing is likely to trigger the release of endoCBs and to regulate glomerular activity. Bursting of neurons may modulate endoCB release not only in the olfactory bulb but also in other brain systems and constitute a general phenomenon of endoCB signaling.

Neuronal activity and signaling in a model neural circuit such as the olfactory bulb glomerulus is regulated by endoCBs in the form of DSI through CB1R-mediated retrograde signaling among glomerular neurons. EndoCBs are synthesized and released from neuronal cell bodies in response to membrane depolarization or cellular excitation [19]. External tufted cells in the glomerular layer can be a potential source of endoCBs. They synapse onto presynaptic cells, *i.e.*, periglomerular cells, and receive GABAergic feedback input. Sensory or synaptic input to external tufted cells can trigger the release of endoCBs and inhibit presynaptic periglomerular cells. This CB1R-mediated inhibition of periglomerular cells reduces their GABA release and, in turn, modifies the firing pattern of external tufted cells. EndoCBs thus reduce inhibitory input to external tufted cells and enhance external tufted cell sensitivity to weak sensory inputs by depolarizing the membrane potential closer to spike threshold. The functional relevance of this signaling pathway lies in a potential increase of the overall sensitivity of the glomerulus to sensory inputs resulting from activation of CB1R on periglomerular cells.

## 6. Cannabinoids and Drug Addiction

The results discussed above indicate that endoCBs function as retrograde messengers to inhibit the activity of neurons that are presynaptic to principal cells, namely periglomerular cells. The inhibition of periglomerular cells through retrograde signaling controls their GABA release and, in turn, regulates principal cell activity. These findings provide novel insights about the function of endoCBs in the olfactory system and by extension of exogenously produced CBs, *i.e.*, marijuana.

Neuroscience drug abuse research attempts to understand the cellular and molecular mechanisms that mediate the transition from occasional, controlled drug use to chronic addiction as shown by a loss of behavioral control over drug seeking and drug taking [1]. Drug addiction is accompanied by modifications in the brain. CBs, *i.e.*, marijuana, derive their addictive properties from powerful within-system neuroadaptations (signal transduction mechanisms) and between-system neuroadaptations (neurocircuitry changes) in the brain motivational and stress systems [1]. An understanding of the cellular mechanisms of CB signaling is pivotal in order to translate the neurobiology of addiction and marijuana substance use disorder to the realm of the pharmacotherapeutic treatment of addiction. Addiction is a biological disorder as shown by advances in our understanding of the brain in the context of drug addiction [61]. The study of the neurobiological mechanisms of addiction has already educated us in terms of how the brain works, particularly in the domains of reward, motivation, and emotions. Drug addiction, formerly known as substance dependence, is a chronically relapsing disorder or disease and is distinct from occasional, controlled, or social use of an abusable drug. As a chronically relapsing disorder, addiction is characterized by (a) a compulsion to seek and take drugs; (b) loss of control over drug intake; and (c) emergence of a negative emotional state (e.g., dysphoria, anxiety, and irratibility) that defines a motivational withdrawal syndrome when access to the drug is prevented [1]. Research supports the hypothesis that addictions are similar to other chronic relapsing disorders, such as diabetes, asthma, and hypertension, in their chronic relapsing nature and treatment efficacy [62].

It is reasonable to state that the initiation of drug abuse is more associated with social and environmental factors, whereas the progression to a substance use disorder is more associated with neurobiological factors such as the cellular mechanisms of action of CBs on neurons. Addictive drugs produce changes in brain circuits that endure long after the person stops taking them. For cannabis, 13.9% of last-year’s users met the criteria for Substance Abuse or Dependence [1].

Translation of research findings into improved health among health disparity populations remains a challenge. Drugs of abuse such as THC can result in chronic addiction by interacting with endogenous neural pathways in the brain such as the endoCB system. CB1R antagonists such as AM251, represent a potentially useful tool not only for blocking the direct reinforcing effects of THC, nicotine, and ethanol, but also for preventing relapse to the use of various drugs of abuse, including cocaine, methamphetamine, and heroin [63]. Clinical and preclinical studies suggest that ligands blocking CB1 receptors offer a novel approach for patients suffering from drug dependence that may be effective across different classes of abused drugs. These studies can lead to a better understanding of drug addiction and pave the way for new pharmacological treatment strategies to reduce craving and addictive behavior.

## 7. Conclusions

Marijuana (cannabis) exhibits neurological and psychiatric effects in the nervous system. In addition to being known and used as recreational drugs, cannabinoids are produced endogenously by neurons in the brain (endocannabinoids, endoCBs) and serve as important signaling molecules in the nervous system and the rest of the body. The most distinguishing feature of endoCBs is their ability to act as retrograde messengers in neural circuits. Here, we reviewed recent advances and findings about the cellular mechanisms and functions of this novel brain signaling system in regulating neural activity at synapses in olfactory circuits. In the olfactory bulb, endoCBs function as retrograde messengers to inhibit the activity of neurons that are presynaptic to principal cells, namely periglomerular cells. The inhibition of periglomerular cells through retrograde signaling controls their GABA release and, in turn, regulates principal cell activity. These studies have the potential to provide the groundwork for translating the neurobiology of drug abuse to the realm of pharmacotherapeutic treatment of addiction. Further studies of retrograde signaling and its regulation in olfactory and other neural circuits can help in developing treatments of marijuana substance use disorder.
